# Centre of pressure characteristics in normal, planus and cavus feet

**DOI:** 10.1186/s13047-018-0245-6

**Published:** 2018-02-05

**Authors:** Andrew K. Buldt, Saeed Forghany, Karl B. Landorf, George S. Murley, Pazit Levinger, Hylton B. Menz

**Affiliations:** 1 La Trobe Sport and Exercise Medicine Research Centre, School of Allied Health, La Trobe University, Melbourne, Victoria 3086 Australia; 2 Discipline of Podiatry, School of Allied Health, La Trobe University, Melbourne, Victoria 3086 Australia; 3 Musculoskeletal Research Center, Isfahan University of Medical Sciences, Isfahan, Iran; 4 Health Sciences Research Centre, University of Salford, Greater Manchester, UK; 5 Institute of Sport, Exercise & Active Living (ISEAL), College of Sport and Exercise Science, Victoria University, Melbourne, Australia

**Keywords:** Foot, Gait, Biomechanics, Foot posture, Centre of pressure

## Abstract

**Background:**

The aim of this study was to compare centre of pressure (COP) characteristics between healthy adults with normal, planus or cavus feet who were allocated to groups based on reliable foot posture measurement techniques.

**Methods:**

Ninety-two healthy adult participants (aged 18 to 45) were recruited and classified as either normal (*n* = 35), pes planus (*n* = 31) or pes cavus (*n* = 26) based on Foot Posture Index, Arch Index and normalised navicular height truncated measurements. Barefoot walking trials were conducted using an emed^®^-x 400 plantar pressure system (Novel GmbH, Munich, Germany). Average, maximum, minimum and range (difference between maximum and minimum) values were calculated for COP velocity and lateral-medial force index during loading response, midstance, terminal stance and pre-swing phases of stance. The COP excursion index was also calculated. One-way analyses of variance were used to compare the three foot posture groups.

**Results:**

The cavus foot exhibited the slowest average and minimum COP velocity during terminal stance, but this pattern was reversed during pre-swing, when the cavus foot exhibited the fastest maximum COP velocity. The planus foot exhibited the smallest lateral medial force index range during terminal stance. There were no differences between the groups for COP excursion index.

**Conclusion:**

These findings indicate that there are differences in COP characteristics between foot postures, which may represent different mechanisms for generating force to facilitate forward progression of the body during the propulsive phases of gait.

## Background

The shape of the human foot during standing, referred to as foot posture, is a commonly measured clinical variable. Recent systematic reviews have found that non-normal foot postures, such as pes planus (low medial longitudinal arch) and pes cavus (high medial longitudinal arch) are associated with increased odds of injury to the lower extremity [1]. In particular, the planus foot has been found to be associated with increased risk of medial tibial stress syndrome and patellofemoral pain [2].

However, the mechanism that links foot posture to lower limb injury is currently unclear, as is the interaction between foot posture and both intrinsic and extrinsic risk factors for lower limb injury. Hence, laboratory-based research has been undertaken to understand the effect of foot posture on foot and lower limb biomechanics. The biomechanical variables that are typically measured include kinematics (motion of body segments), electromyography (muscle activity) and plantar pressure measurement (force applied to the plantar surface of the foot) [3].

An important subgroup of plantar pressure measurement is the centre of pressure (COP), also referred to as the ‘gait line’. The COP is defined as the centroid of the total number of active sensors for each data sample collected, which represents the spatial distribution of pressure over time [4]. As a global measure of pressure distribution, it has been suggested that the COP provides greater insight into dynamic foot function compared to measures that are confined to discrete regions, such as peak pressure and maximum force [5].

There are two studies that have compared COP between all 3 foot postures (normal, planus and cavus) [5, 6], and one study that compared planus and normal feet [7]. Of these studies, two characterised the COP using the COP excursion index, which involves the measurement of the deviation of the COP from a reference line as it traverses the anterior third of the foot [6, 7]. The COP excursion index has been widely used to investigate foot function. For example, the COP excursion index has been used to examine specific populations, such as older adults [8], and specific pathologies, such as hallux valgus [9]. The foot posture studies have found less concave trajectory (indicative of greater medial deviation) of the COP in the planus foot compared to normal and cavus feet [6, 7]. This suggests comparatively greater force being borne on the medial plantar surface of the planus foot. However, a limitation of these studies was that foot posture groups were assigned using angular measurements of foot alignment, such as the resting calcaneal stance position, which has been found to have poor reliability [10, 11]. Hence, there is a need to compare the COP excursion index using reliable foot posture measurement approaches with normative data.

Furthermore, the available literature does not allow for definitive conclusions to be drawn regarding how spatial and temporal characteristics of the COP may differ between foot postures throughout the entirety of the stance phase. Measures that are able to describe the dynamic characteristics of the COP in the literature include the velocity of the COP [4] and the lateral-medial force index [12]. Although one investigation using these measures compared individuals with different foot postures during running [13], they did not analyse overground walking.

With this in mind, the aim of this study was to compare COP characteristics between normal, planus and cavus foot posture groups that were assigned using reliable foot posture measurement techniques supported with normative data. The COP measures for comparison include the velocity of the COP, the lateral-medial force index and the COP excursion index.

## Methods

### Participants

Ninety-two healthy adult participants (aged 18 to 45) were recruited from the general student and staff population of La Trobe University via posters that were placed around the university campus. Volunteers were excluded from the study if they reported any current or recurring musculoskeletal lower limb injury or any neurovascular condition or biomechanical abnormality that may affect gait. A screening protocol was carried out to measure foot posture on both feet and assign participants to groups using the 6-item Foot Posture Index (FPI) [14], the Arch Index (AI) [15], and normalised navicular height truncated (NNHt) [16]. Participants qualified for the normal group if the static foot measurements were within one standard deviation of the mean of normative data for the FPI [14] and either the AI or NNHt [17]. Participants were assigned to the pes cavus or pes planus group if static foot measurements were greater or less than one standard deviation from the mean of normative data for the FPI and either the AI or NNHt. Boundaries for the inclusion into foot posture groups are shown in Table 1.

**Table 1:** Boundaries for the inclusion into foot posture groups

Foot posture measurement	Normal	Pes planus	Pes cavus
FPI	+ 1 to + 7	> + 7	< + 1
Arch Index	0.11 to 0.25	> 0.25	< 0.11
Normalised navicular height truncated	0.31 to 0.22	< 0.22	> 0.31

One foot of each participant was selected for testing and analysis. If both feet satisfied the selection criteria, one foot was randomly selected for testing (using the random number generator function in Microsoft Excel^®^ Microsoft Corporation, Redmond,WA). Otherwise, if only one foot satisfied the selection criteria for a group, then this foot was tested. There was one instance when this occurred and the participant was allocated to the normal group. Ethical approval was granted by the La Trobe University Human Ethics Committee (ID number: HEC11-097) and all participants signed informed consent.

### Instrumentation

Dynamic barefoot plantar pressure data were collected using an emed^®^-x 400 plantar pressure system (Novel GmbH, Munich, Germany), a 700 mm long by 403 mm wide platform incorporating 6080 capacitance transducer sensors (4 sensors/cm^2^) sampling at a frequency of 100 Hz. The platform was embedded in the centre of a flat, gait analysis laboratory walkway.

### Experimental protocol

After a five-minute acclimatisation period, participants were instructed to walk along the walkway using the two-step initiation protocol. Participants were positioned two steps from the front edge of the platform and instructed to walk at their comfortable walking speed [18]. The two-step initiation protocol was used as it requires fewer trials to be collected compared to the midgait protocol because it is less likely that participants will miss contacting the pressure platform. In addition, it has been shown that two steps is sufficient to reach steady state walking [19] and it has equivalent reliability to the midgait protocol [20, 21]. Participants were asked not to look at the ground during walking trials, and in the event of targeting of the pressure platform, the trial was not analysed. Five successful trials were analysed.

### Data processing and statistical analysis

**Fig. 1:**
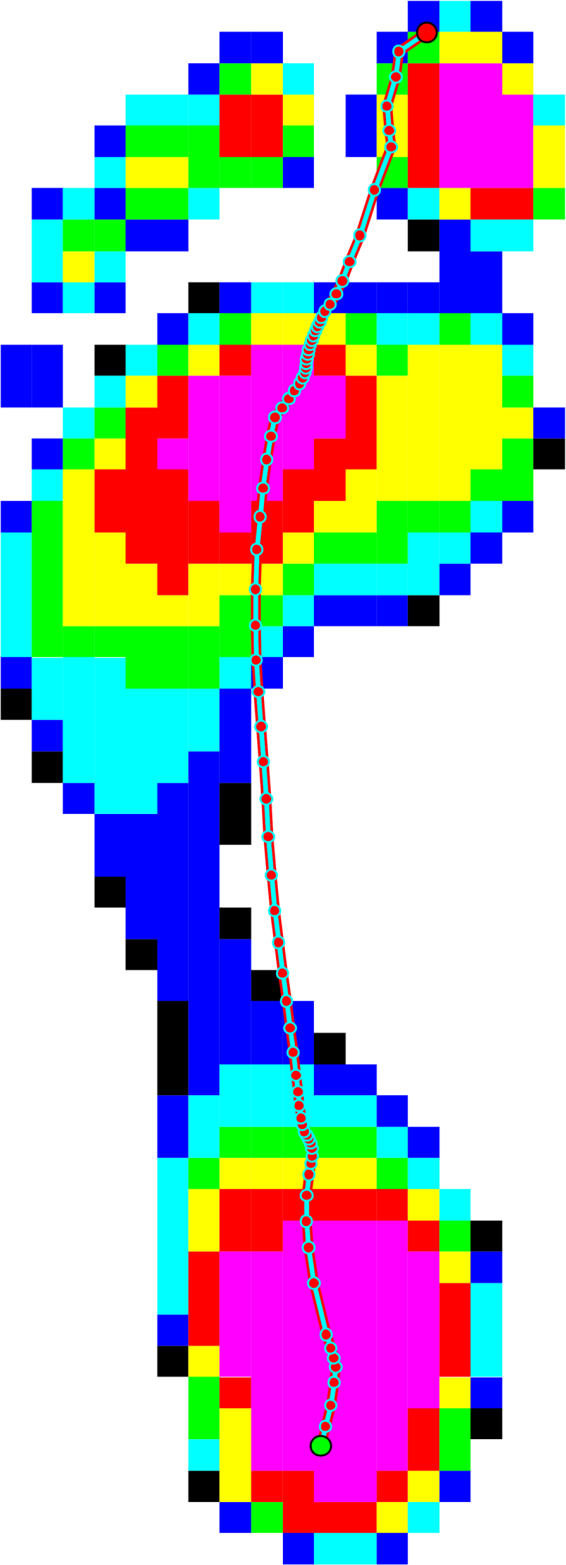
Typical centre of pressure distribution

The COP, defined as the centroid of the total number of active sensors for each data sample collected (Fig. 1) was the main parameter of interest [4]. The variables that were derived from the COP were as follows:

(i) The velocity of the COP (m/s), defined as the resultant displacement of the COP divided by the elapsed time between measurements [4]
(ii) The lateral-medial force index, calculated as the amount of force lateral to the COP divided by the amount of force medial to the COP [22]
(iii) The COP excursion index, defined as the excursion of the COP from a constructed line connecting the first and the last points of the COP curve measured at the distal third of the foot and normalised to foot width [7]

Variables were obtained for each participant using the Novel Scientific Medical Software, Version 23. Both the velocity of the COP and the lateral-medial force index were normalised to stance period duration. Stance period was then divided into four phases: (i) loading response (0 to 20% of stance), (ii) midstance (> 20 to 52%), (iv) terminal stance (> 52 to 83%), and (v) pre-swing (> 83 to 100%) [23]. For each of the four phases of stance, the average, maximum, minimum and range (difference between maximum and minimum values) were calculated for each participant.

All statistical tests were calculated using SPSS version 24 for Windows (IBM Corporation, NY). The distribution of data for all groups was assessed for skewness, kurtosis and equality of variance (Levene’s test). A one-way analysis of variance (ANOVA) was performed with significance level set at < 0.05 with Bonferroni adjustment (α = 0.017). Post-hoc comparison of the mean differences between groups was applied to all ANOVAs. Confidence intervals and effect sizes using Cohen’s *d* were calculated for all significant mean differences. The following interpretation of effect size was used: trivial: 0-0.2, small: 0.2-0.6, moderate: 0.6-1.2 and large: 1.2-2.0 [24].

## Results

### Participant characteristics

All anthropometric and foot posture characteristics are shown in Table 2. The normal foot posture group consisted of 35 participants (17 male, 18 female), the pes planus group consisted of 31 participants (16 male, 15 female) and pes cavus group consisted of 26 participants (12 male, 14 female). The height of the planus group was significantly less than the normal and cavus groups. Even though such a difference may influence COP by influencing step length, and therefore walking velocity it is unlikely as there was no difference in total contact time, a proxy measure of walking velocity [25]. Therefore, no adjustment was made for height. No further variables were significantly different other than foot posture classification measures.

**Table 2:** Participant characteristics mean (SD) unless otherwise stated

	Normal (n = 35)	Planus (n = 31)	Cavus (n = 26)
Anthropometric measures			
Sex, n = M/F	17/18	16/15	12/14
Age, years	25.1 (5.1)	24.5 (5.9)	26.1 (7.2)
Height, cm	172.9 (9.3)	166.9 (9.7)	173.7 (11.1)
Weight, kg	70.0 (13.7)	69.2 (17.1)	72.4 (14.3)
BMI, kg/m^2^	23.4 (3.2)	24.6 (5.1)	23.7 (2.7)
Foot posture measures			
FPI	3.8 (1.0), range: 1-6	9.0 (1.0), range 8-12	−1.4 (1.2), range: −4-0
NNHt	0.25 (0.02), range: 0.20-0.32	0.19 (0.03), range: 0.11-0.24	0.32 (0.02), range: 0.29-0.37
AI	0.22 (0.03), range: 0.15-0.29	0.30 (0.05), range: 0.23-0.40	0.16 (0.06), range: 0.04-0.24
Contact time, ms	698.8 (52.1)	699.8 (68.9)	707.7 (66.2)

*FPI* Foot Posture Index, *NNHt* normalised navicular height truncated, *AI* Arch Index

### Velocity of the COP

**Fig. 2:**
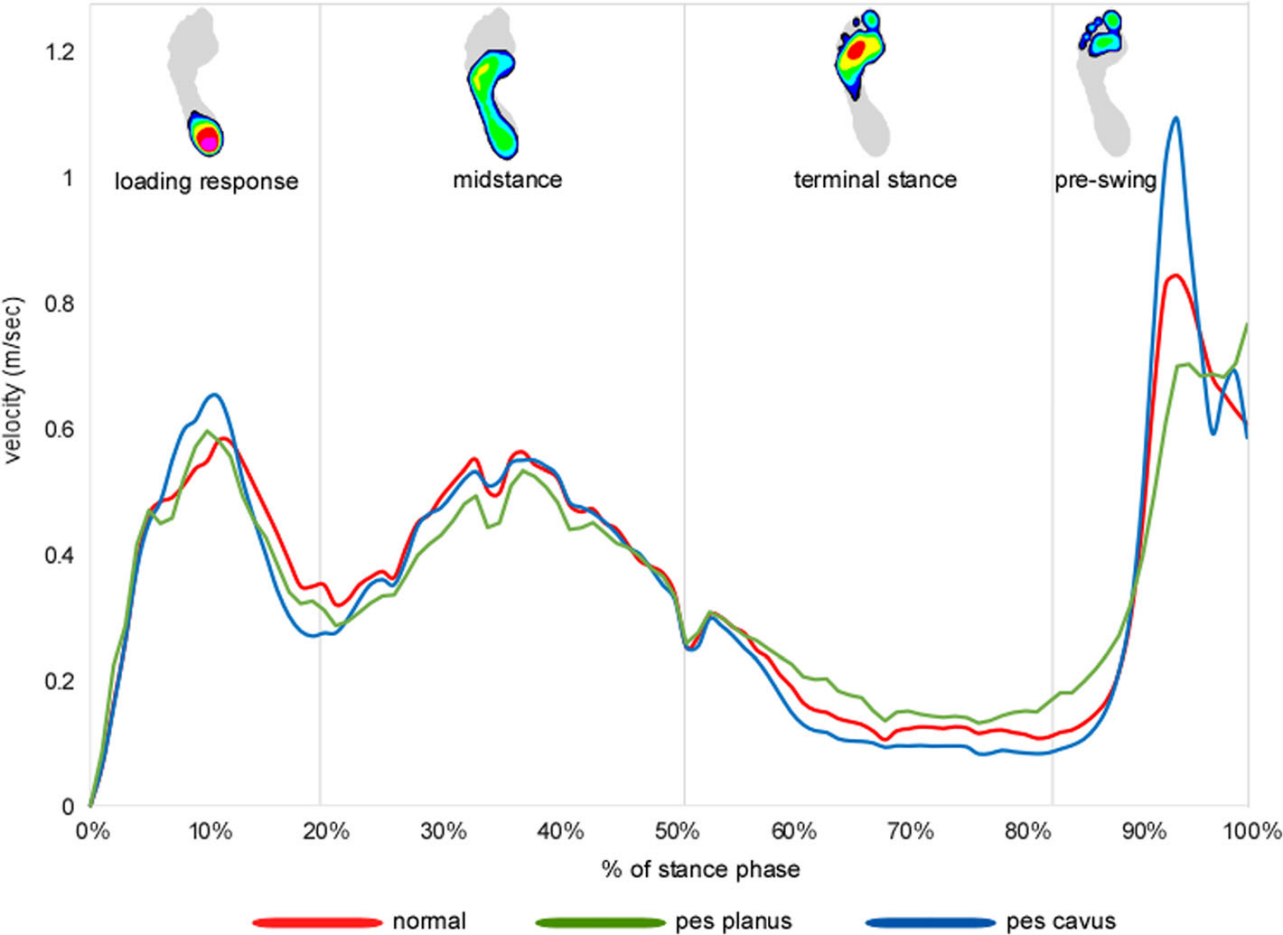
Ensemble average for the centre of pressure velocity during stance phase for normal, planus and cavus feet

A graphical representation of the ensemble average for velocity of the COP during stance phase is shown in Fig. 2, and all comparisons between group means with 95% confidence intervals for velocity of the COP are shown in Table 3. The cavus foot exhibited the slowest average and minimum COP velocity during terminal stance compared to the planus foot, but this pattern was reversed during pre-swing, with the cavus foot exhibiting the fastest maximum COP velocity. Effect sizes for these comparisons ranged from 0.66 (moderate) to 1.49 (large).

**Table 3:** Centre of pressure velocity (m/s) during each stance period phase for each of the foot posture groups

	Normal mean (SD)	Planus mean (SD)	Cavus mean (SD)	Planus vs normal mean difference (95% CI) ES^a^	Cavus vs normal mean difference (95% CI) ES^a^	Planus vs cavus mean difference (95% CI) ES^a^
Loading response						
Average	0.405 (0.084)	0.397 (0.070)	0.400 (0.082)	−0.008 (− 0.056 to 0.040)	−0.005 (− 0.059 to 0.046)	− 0.003 (− 0.055 to 0.0482)
Maximum	0.845 (0.245)	0.797 (0.220)	0.909 (0.272)	− 0.048 (− 0.198 to 0.102)	0.064 (− 0.091 to 0.220)	− 0.112 (− 0.273 to 0.048)
Minimum	0.000 (0.000)	0.000 (0.000)	0.000 (0.000)	0.000 (0.000 to 0.000)	0.000 (0.000 to 0.000)	0.000 (0.000 to 0.000)
Range	0.854 (0.247)	0.795 (0.223)	0.909 (0.267)	−0.050 (− 0.199 to 0.099)	0.064 (− 0.090 to 0.218)	− 0.114 (− 0.272 to 0.045)
Midstance						
Average	0.435 (0.061)	0.403 (0.091)	0.442 (0.119)	−0.032 (− 0.088 to 0.024)	0.007 (− 0.051 to 0.065)	−0.039 (− 0.098 to 0.020)
Maximum	0.648 (0.124)	0.596 (0.144)	0.641 (0.161)	−0.052 (− 0.139 to 0.035)	−0.008 (− 0.097 to 0.081)	−0.044 (− 0.136 to 0.048)
Minimum	0.163 (0.065)	0.163 (0.059)	0.173 (0.192)	0.000 (−0.072 to 0.071)	0.010 (−0.064 to 0.083)	0.010 (− 0.086 to 0.065)
Range	0.485 (0.157)	0.434 (0.159)	0.486 (0.166)	−0.051 (− 0.149 to 0.047)	0.002 (− 0.100 to 0.103)	−0.052 (− 0.158 to 0.052)
Terminal stance						
Average	0.177 (0.069)	0.200 (0.063)	0.152 (0.056)	0.023 (−0.016 to 0.062)	−0.025 (− 0.066 to 0.015)	0.048 (0.006 to 0.090) 0.86^b^
Maximum	0.384 (0.144)	0.386 (0.133)	0.361 (0.149)	0.001 (−0.085 to 0.088)	− 0.022 (− 0.113 to 0.068)	0.024 (− 0.068 to 0.117)
Minimum	0.071 (0.030)	0.098 (0.039)	0.050 (0.024)	0.027 (0.008 to 0.046) 0.77^b^	−0.021 (− 0.041 to − 0.001) 0.78^b^	0.049 (0.028 to 0.069) 1.49^c^
Range	0.312 (0.133)	0.287 (0.127)	0.311 (0.136)	−0.026 (− 0.106 to 0.055)	−0.001 (− 0.085 to 0.083)	−0.024 (− 0.111 to 0.062)
Pre-swing						
Average	0.453 (0.098)	0.451 (0.082)	0.484 (0.074)	−0.002 (− 0.055 to 0.051)	0.031 (− 0.024 to 0.086)	− 0.033 (− 0.090 to 0.023)
Maximum	1.324 (0.450)	1.197 (0.493)	1.520 (0.487)	− 0.127 (− 0.418 to 0.164)	0.193 (− 0.110 to 0.495)	− 0.319 (− 0.631 to − 0.008) 0.66^b^
Minimum	0.092 (0.052)	0.141 (0.066)	0.078 (0.040)	− 0.049 (− 0.083 to − 0.016) 0.82^b^	0.014 (− 0.021 to 0.048)	− 0.063 (− 0.098 to − 0.027) 1.15^c^
Range	1.448 (0.412)	1.333 (0.502)	1.595 (0.473)	− 0.115 (− 0.402 to 0.172)	0.146 (− 0.149 to 0.442)	−0.261 (− 0.566 to 0.043)

^a^ ES: Effect size (Cohen’s *d*) only reported for statistically significant comparisons. *CI* confidence interval

^b^ Significant difference between groups, *p* < 0.05

^c^ Significant difference between groups, *p* < 0.01

### Lateral-medial force index

**Fig. 3:**
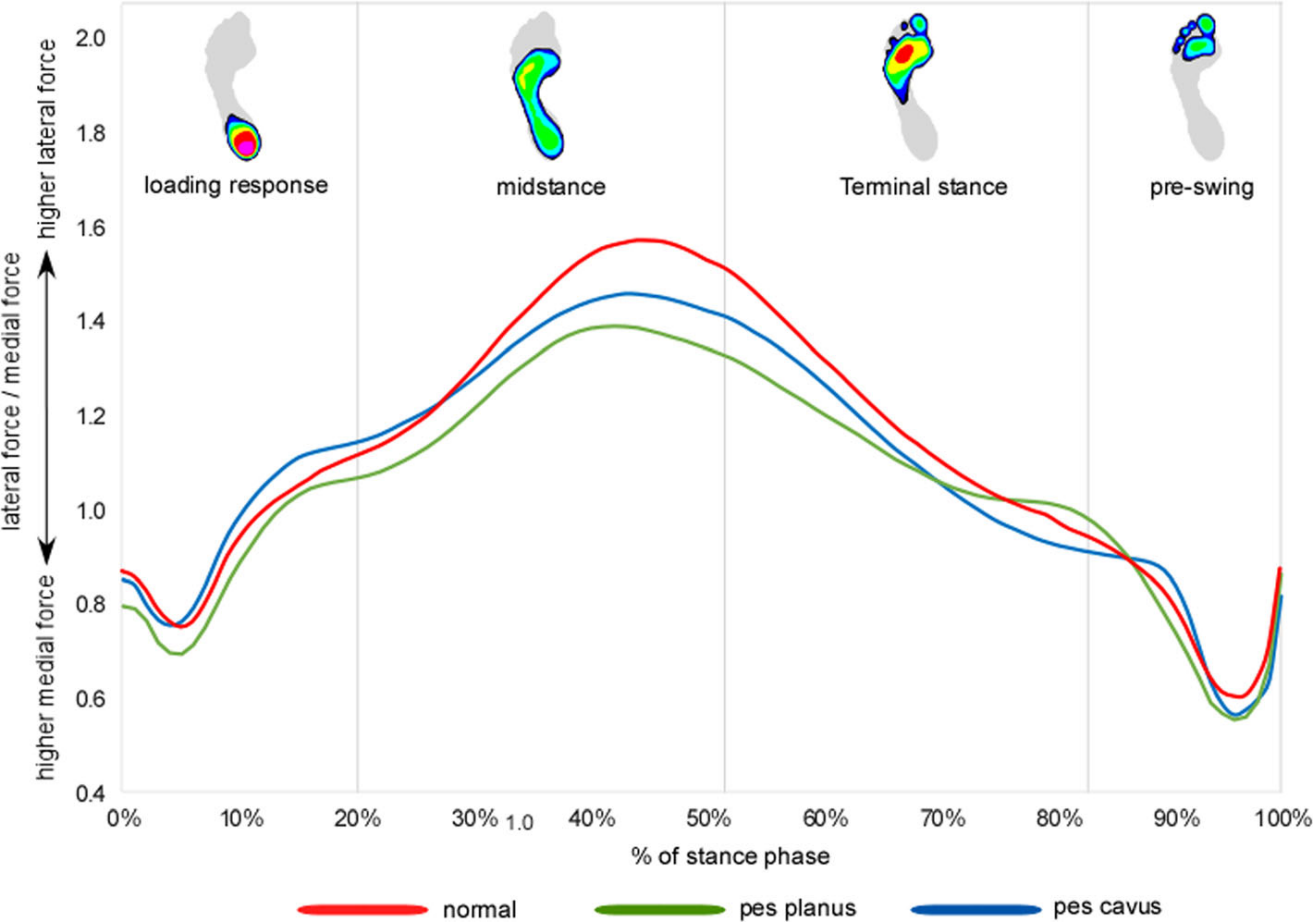
Ensemble average for the centre of pressure lateral–medial force index for normal, planus and cavus feet

A graphical representation of the ensemble averages for the lateral-medial force index during stance phase is shown in Fig. 3, and all comparisons between group means with 95% confidence intervals and effect sizes for the lateral-medial force index are shown in Table 4. During terminal stance, the planus foot exhibited a lower range compared to the normal foot. The effect size for this comparison was 0.65 (moderate).

**Table 4:** Lateral-medial force index during each stance period phase for each of the foot posture groups. Higher values represent higher relative force lateral to the centre of pressure

	Normal mean (SD)	Planus mean (SD)	Cavus mean (SD)	Planus vs normal mean difference (95% CI) ES^a^	Cavus vs normal mean difference (95% CI) ES^a^	Planus vs cavus mean difference (95% CI) ES^a^
Loading response						
Average	0.945 (0.142)	0.887 (0.116)	0.978 (0.206)	− 0.058 (− 0.156 to 0.039)	0.033 (− 0.068 to 0.134)	−0.091 (− 0.194 to 0.012)
Maximum	1.169 (0.199)	1.164 (0.255)	1.289 (0.353)	−0.005 (− 0.176 to 0.167)	0.119 (− 0.057 to 0.296)	−0.124 (− 0.304 to 0.055)
Minimum	0.711 (0.181)	0.631 (0.207)	0.715 (0.221)	−0.079 (− 0.206 to 0.046)	0.004 (− 0.126 to 0.134)	−0.084 (− 0.217 to 0.050)
Range	0.507 (0.220)	0.523 (0.275)	0.573 (0.281)	0.016 (−0.144 to 0.175)	0.066 (− 0.100 to 0.233)	− 0.050 (− 0.219 to 0.118)
Midstance						
Average	1.400 (0.249)	1.276 (0.161)	1.343 (0.199)	−0.124 (− 0.255 to 0.008)	−0.056 (− 0.192 to 0.079)	−0.067 (− 0.208 to 0.073)
Maximum	1.592 (0.263)	1.450 (0.207)	1.561 (0.259)	−0.142 (− 0.297 to 0.012)	−0.032 (− 0.192 to 0.128)	−0.111 (− 0.275 to 0.054)
Minimum	1.077 (0.141)	1.025 (0.178)	1.070 (0.189)	−0.052 (− 0.158 to 0.054)	−0.007 (− 0.117 to 0.103)	−0.044 (− 0.157 to 0.068)
Range	0.527 (0.295)	0.405 (0.173)	0.493 (0.238)	−0.122 (− 0.274 to 0.029)	−0.033 (− 0.189 to 0.123)	−0.089 (− 0.249 to 0.072)
Terminal stance						
Average	1.235 (0.204)	1.153 (0.115)	1.148 (0.157)	−0.082 (− 0.186 to 0.022)	−0.087 (− 0.193 to 0.019)	0.005 (− 0.105 to 0.115)
Maximum	1.474 (0.193)	1.383 (0.139)	1.437 (0.262)	−0.090 (− 0.219 to 0.038)	−0.036 (− 0.168 to 0.095)	−0.054 (− 0.189 to 0.080)
Minimum	0.941 (0.189)	0.954 (0.163)	0.891 (0.149)	0.013 (−0.092 to 0.118)	−0.049 (− 0.158 to 0.059)	0.063 (− 0.049 to 0.175)
Range	0.617 (0.372)	0.426 (0.183)	0.546 (0.281)	−0.190 (− 0.375 to − 0.006) 0.65^b^	−0.070 (− 0.259 to 0.118)	−0.120 (− 0.317 to 0.077)
Pre-swing						
Average	0.838 (0.186)	0.772 (0.151)	0.782 (0.121)	−0.065 (− 0.165 to 0.033)	−0.056 (− 0.158 to 0.047)	−0.009 (− 0.116 to 0.096)
Maximum	1.118 (0.208)	1.120 (0.241)	1.055 (0.121)	0.002 (−0.126 to 0.131)	−0.063 (− 0.195 to 0.069)	0.065 (− 0.068 to 0.200)
Minimum	0.513 (0.136)	0.459 (0.102)	0.494 (0.124)	0.054 (−0.024 to 0.132)	0.019 (−0.061 to 0.098)	0.035 (− 0.046 to 0.116)
Range	1.631 (0.243)	1.579 (0.263)	1.538 (0.167)	−0.052 (− 0.200 to 0.097)	−0.093 (− 0.246 to 0.060)	0.041 (− 0.114 to 0.196)

*ES* effect size, CI: confidence interval

^a^ Effect size (Cohen’s *d*) only reported for statistically significant comparisons

^b^ Significant difference between groups, *p* < 0.05

### Centre of pressure excursion index

For the COP excursion index, all comparisons between group means with 95% confidence intervals are shown in Table 5. There were no significant differences for any comparison between groups.

**Table 5:** Centre of pressure excursion index and comparisons of all foot postures. Lower values represent less concave trajectory (greater medial deviation) of the COP

Normal mean (SD)	Planus mean (SD)	Cavus mean (SD)	Planus vs normal mean difference (95% CI)	Cavus vs normal mean difference (95% CI)	Planus vs cavus mean difference (95% CI)
20.4 (6.5)	18.4 (4.5)	20.2 (5.8)	−2.0 (− 5.4 to 1.4)	−0.2 (− 3.8 to 3.4)	−1.8 (− 5.5 to 1.8)

*CI* confidence interval

## Discussion

The aim of this study was to compare COP characteristics between normal, planus and cavus foot posture groups that were assigned using reliable foot posture measurement techniques supported with normative data. Overall, few differences in COP characteristics were observed between the groups. The most notable difference between foot postures was in relation to COP velocity during the terminal stance and pre-swing phases of the gait cycle. Effect sizes for these significant differences were medium to large (ranging between 0.65 and 1.49), which are comparable to previous research comparing COP between foot postures [6, 7].

The velocity of the COP for all 3 foot postures reflected the shape and magnitude of the normative description using healthy uninjured individuals that was reported by Cornwall and McPoil [4]. However, significant differences between all 3 foot postures were found for COP velocity during terminal stance and pre-swing. Specifically, during terminal stance, significant differences for the minimum value of COP velocity were found between the planus, normal and cavus foot posture groups in a manner that would suggest a dose-response relationship across the spectrum of foot postures. For example, the cavus foot demonstrated the slowest (lowest minimum value) COP velocity, while the planus group demonstrated the fastest COP velocity in terminal stance. However, during pre-swing, this relationship was reversed, in that the cavus foot demonstrated the highest maximum COP velocity.

The differences in COP velocity has implications for foot function. For example, during terminal stance, the forefoot is the only structure in contact with the ground. Therefore, sufficient force must be generated to drive the lower limb forward over the forefoot, otherwise gravity would prevent the forward progression of the body [26]. The generation of force required is associated with slowing of the velocity of the COP as it traverses the metatarsophalangeal joints.

Differences in the velocity of the COP during terminal stance suggest subtle variations between foot postures in the mechanism for generating force that is required for forward progression. Specifically, the direction of the force vector that is applied to the forefoot may be influenced by differences in skeletal positioning (i.e. greater comparative plantarflexion of the forefoot in the cavus foot compared to the planus foot) or joint characteristics (i.e. greater rigidity for a given amount of force in the cavus foot compared to the planus foot) [27]. In cavus feet, the force vector may have a greater vertical component compared to normal and planus feet, causing the anterior-posterior movement of the COP to progress more slowly. Conversely, in planus feet, the force vector may contain a comparatively greater anterior-posterior component, thus allowing the COP to advance more rapidly.

During pre-swing, as the contra-lateral foot strikes the ground, both gravity and the generation of force by posterior leg muscles contribute to the forward propulsion of the body over the metatarsophalangeal joints [28]. During pre-swing, the velocity of the COP rapidly increases to peak during stance. In the cavus foot, the peak value of COP velocity was higher than in the planus foot. This finding indicates that the strategy to generate force during terminal stance creates adequate angular momentum to produce efficient forward progression of the body [4]. In contrast, for the planus foot, COP velocity was slowed. This may be a result of greater force and pressure being borne on the hallux, which acts as a barrier to forward progression of the COP. While there was no evidence in this study of a medial shift of the COP during pre-swing in the planus foot, previous plantar pressure studies have shown that in the planus foot, greater pressure and force is borne by the hallux [6, 29]. Greater loading of the hallux and associated slowing of the COP has also been demonstrated in a study that compared participants with osteoarthritis of the 1st metatarsophalangeal joint to healthy controls [30]. The slowed COP in the planus foot may also be in response to a delay in the establishment of tensile forces in plantar tissues of the foot due to first metatarsophalangeal joint dorsiflexion. This phenomenon, referred to as the windlass mechanism, is understood to allow for greater stiffness in the joint of the midfoot and forefoot during propulsion [31]. While some kinematic studies support our suggestion of a link between foot posture and establishment of the windlass mechanism, further work is required to substantiate this proposed association [29, 32].

The only significant finding not related to COP velocity was during terminal stance, where the planus foot exhibited a lower range of the lateral-medial force index compared to the normal foot. This finding suggests that in the planus foot, structures may be exposed to more repetitive loads and therefore, increased risk of injury as the balance of load either side of the COP remains relatively static. This theory is supported by some studies that suggest a link between repetitive low level loading and the development of lower limb injuries in runners [33, 34].

Despite a trend towards a smaller COP excursion index value in the planus group compared to both normal or cavus foot groups, our study found no significant difference in the COP excursion index between foot posture groups. This differs from similar studies that reported a smaller COP excursion index (indicative of a greater medial deviation of the COP) in the planus foot compared to normal and cavus feet [6, 7]. However, the COP excursion index may not adequately detect differences in the location and the progression of the COP as they occur throughout the entirety of stance due to it measuring the medio-lateral location of the COP at a single site on the forefoot (the anterior third trisection of the foot) [6]. Therefore, the COP excursion index does not provide any insight into the location of the COP posterior and anterior to the measurement location. Further work is needed to determine the most beneficial method for reporting the characteristics of the COP throughout the entirety of stance.

There are several limitations to this study. Firstly, the plantar pressure measurement system used in this study can only detect vertical force; other forces that may be relevant to the movement of forces on the plantar foot, such as shear force, are not detected. Secondly, associated biomechanical data, such as kinematics and electromyography were not collected, so it is difficult to make conclusions about the influence of foot posture on overall foot function. Thirdly, the cavus group included feet with a narrower range of foot postures in comparison to the planus group, which may have limited our ability to detect differences between the groups. Finally, all analyses were conducted unshod, and it is likely that footwear characteristics, such as sole bending stiffness, may influence the COP while wearing shoes.

## Conclusion

This study found an overall similarity in the characteristics of the COP between normal, planus and cavus foot posture groups. Of the differences that were found, the most notable were in the velocity of the COP during the terminal stance and pre-swing phases of gait that may represent different mechanisms for generating force to accomplish forward progression of the body. A difference was also found between the planus and normal foot in relation to the medial shift of the lateral-medial force index during terminal stance. These findings add to the understanding of how foot posture affects the COP, particularly during the propulsive phases of gait. Further research is needed to determine whether there is a relationship between variation in COP and the development of lower limb injury.

## Abbreviations

AI: Arch Index
COP: Centre of pressure
FPI: Foot Posture Index
NNHt: Normalised navicular height truncated

## References

[1] Tong JW, Kong PW (2013). Association between foot type and lower extremity injuries: systematic literature review with meta-analysis. J Orthop Sports Phys Ther.

[2] Neal BS, Griffiths IB, Dowling GJ, Murley GS, Munteanu SE, Franettovich Smith MM, et al. Foot posture as a risk factor for lower limb overuse injury: a systematic review and meta-analysis. J Foot Ankle Res. 2014;7:55. 10.1186/s13047-014-0055-4 PMC4282737 25558288

[3] Landorf KB, Keenan A-M (2000). Efficacy of foot orthoses. What does the literature tell us?. J Am Podiatr Med Assoc.

[4] Cornwall MW, McPoil TG (2000). Velocity of the center of pressure during walking. J Am Podiatr Med Assoc.

[5] Wong L, Hunt A, Burns J, Crosbie J (2008). Effect of foot morphology on center-of-pressure excursion during barefoot walking. J Am Podiatr Med Assoc.

[6] Hillstrom HJ, Song J, Kraszewski AP, Hafer JF, Mootanah R, Dufour AB (2013). Foot type biomechanics part 1: structure and function of the asymptomatic foot. Gait Posture..

[7] Song J, Hillstrom HJ, Secord D, Levitt J (1996). Foot type biomechanics. Comparison of planus and rectus foot types. J Am Podiatr Med Assoc.

[8] Hagedorn TJ, Dufour AB, Golightly YM, Riskowski JL, Hillstrom HJ, Casey VA, et al. Factors affecting center of pressure in older adults: the Framingham foot study. J Foot Ankle Res. 2013;6:18. 10.1186/1757-1146-6-18 PMC3655877 23657058

[9] Galica AM, Hagedorn TJ, Dufour AB, Riskowski JL, Hillstrom HJ, Casey VA, et al. Hallux valgus and plantar pressure loading: the Framingham foot study. J Foot Ankle Res. 2013;6:42. 10.1186/1757-1146-6-42 PMC3819471 24138804

[10] Jarvis HL, Nester CJ, Jones RK, Williams A, Bowden PD. Inter-assessor reliability of practice based biomechanical assessment of the foot and ankle. J Foot Ankle Res. 2012;5:14. 10.1186/1757-1146-5-14 PMC3431260 22716130

[11] Menz HB, Keenan A-M (1997). Reliability of two instruments in the measurement of closed chain subtalar joint positions. Foot (Edinb)..

[12] Cornwall MW, McPoil TG (2003). Reliability and validity of center-of-pressure quantification. J Am Podiatr Med Assoc.

[13] De Cock A, Vanrenterghem J, Willems T, Witvrouw E, De Clercq D (2008). The trajectory of the centre of pressure during barefoot running as a potential measure for foot function. Gait Posture..

[14] Redmond AC, Crane YZ, Menz HB. Normative values for the foot posture index. J Foot Ankle Res. 2008;1:6. 10.1186/1757-1146-1-6 PMC2553778 18822155

[15] Cavanagh PR, Rodgers MM (1987). The arch index: a useful measure from footprints. J Biomech.

[16] Cowan DN, Jones BH, Robinson JR (1993). Foot morphologic characteristics and risk of exercise-related injury. Arch Fam Med.

[17] Murley GS, Menz HB, Landorf KB. A protocol for classifying normal- and flat-arched foot posture for research studies using clinical and radiographic measurements. J Foot Ankle Res. 2009;2:22. 10.1186/1757-1146-2-22 PMC3583243 19575811

[18] Meyers-Rice B, Sugars L, McPoil T, Cornwall M (1994). Comparison of three methods for obtaining plantar pressures in nonpathologic subjects. J Am Podiatr Med Assoc.

[19] Breniere Y, Do MC (1986). When and how does steady state gait movement induced from upright posture begin?. J Biomech.

[20] Putti AB, Arnold GP, Cochrane LA, Abboud RJ. Normal pressure values and repeatability of the Emed^®^ ST4 system. Gait Posture. 2008;27(3):501–5. 10.1016/j.gaitpost.2007.06.009 17702582

[21] Gurney JK, Kersting UG, Rosenbaum D (2008). Between-day reliability of repeated plantar pressure distribution measurements in a normal population. Gait Posture..

[22] Novel. Novel scientific medical manual: Munich; 2012.

[23] Perry J (2010). Gait analysis: normal and pathological function.

[24] Cohen JW (1988). Statistical power analysis for the behavioural sciences (2nd edn).

[25] Taylor AJ, Menz HB, Keenan A-M (2004). The influence of walking speed on plantar pressure measurements using the two-step gait initiation protocol. Foot (Edinb).

[26] Miyazaki S, Yamamoto S (1993). Moment acting at the metatarsophalangeal joints during normal barefoot level walking. Gait Posture..

[27] Aminian A, Sangeorzan BJ (2008). The anatomy of Cavus foot deformity. Foot Ankle Clin.

[28] Zajac FE, Neptune RR, Kautz SA (2002). Biomechanics and muscle coordination of human walking: part I: introduction to concepts, power transfer, dynamics and simulations. Gait Posture..

[29] Rao S, Song J, Kraszewski A, Backus S, Ellis SJ, Md JTD (2011). The effect of foot structure on 1st metatarsophalangeal joint flexibility and hallucal loading. Gait Posture.

[30] Zammit GV, Menz HB, Munteanu SE, Landorf KB (2008). Plantar pressure distribution in older people with osteoarthritis of the first metatarsophalangeal joint (hallux limitus/rigidus). J Orthop Res.

[31] Fuller EA (2000). The windlass mechanism of the foot. A mechanical model to explain pathology. J Am Podiatr Med Assoc.

[32] Lucas R, Cornwall M (2017). Influence of foot posture on the functioning of the windlass mechanism. Foot.

[33] Kaufman KR, Brodine SK, Shaffer RA, Johnson CW, Cullison TR (1999). The effect of foot structure and range of motion on musculoskeletal overuse injuries. Am J Sports Med.

[34] Hreljac A, Marshall RN, Hume PA (2000). Evaluation of lower extremity overuse injury potential in runners. Med Sci Sports Exerc.

